# Estimating individual exposure to predation risk in group-living baboons, *Papio anubis*

**DOI:** 10.1371/journal.pone.0287357

**Published:** 2023-11-08

**Authors:** Alexandre Suire, Itsuki Kunita, Roi Harel, Margaret Crofoot, Mathew Mutinda, Maureen Kamau, James M. Hassel, Suzan Murray, Shoji Kawamura, Akiko Matsumoto-Oda

**Affiliations:** 1 Faculty of Global and Regional Studies, University of the Ryukyus, Okinawa, Japan; 2 Faculty of Engineering, University of the Ryukyus, Okinawa, Japan; 3 Department for the Ecology of Animal Societies, Max Planck Institute of Animal Behavior, Konstanz, Germany; 4 Department of Biology, University of Konstanz, Konstanz, Germany; 5 Kenya Wildlife Service, Nairobi, Kenya; 6 Smithsonian Conservation Biology Institute, Global Health Program, Washington, DC, United States of America; 7 Mpala Research Centre, Nanyuki, Kenya; 8 Graduate School of Frontier Sciences, The University of Tokyo, Chiba, Japan; 9 Graduate School of Tourism Sciences, University of the Ryukyus, Okinawa, Japan; Anglia Ruskin University, UNITED KINGDOM

## Abstract

In environments with multiple predators, vulnerabilities associated with the spatial positions of group-living prey are non-uniform and depend on the hunting styles of the predators. Theoretically, coursing predators follow their prey over long distances and attack open areas, exposing individuals at the edge of the group to predation risk more than those at the center (marginal predation). In contrast, ambush predators lurk unnoticed by their prey and appear randomly anywhere in the group; therefore, isolated individuals in the group would be more vulnerable to predators. These positions of vulnerability to predation are expected to be taken by larger-bodied males. Moreover, dominant males presumably occupy the center of the safe group. However, identifying individuals at higher predation risk requires both simultaneous recording of predator location and direct observation of predation events; empirical observations leave ambiguity as to who is at risk. Instead, several theoretical methods (predation risk proxies) have been proposed to assess predation risk: (1) the size of the individual ‘unlimited domain of danger’ based on Voronoi tessellation, (2) the size of the ‘limited domain of danger’ based on predator detection distance, (3) peripheral/center position in the group (minimum convex polygon), (4) the number and direction of others in the vicinity (surroundedness), and (5) dyadic distances. We explored the age-sex distribution of individuals in at-risk positions within a wild baboon group facing predation risk from leopards, lions, and hyenas, using Global Positioning System collars. Our analysis of the location data from 26 baboons revealed that adult males were consistently isolated at the edge of the group in all predation risk proxies. Empirical evidence from previous studies indicates that adult male baboons are the most frequently preyed upon, and our results highlights the importance of spatial positioning in this.

## Introduction

Animals are often tightly grouped under the threat of predation, with all group members benefiting from reduced predation risk through several mechanisms, including dilution, many-eyes, and confusion effects [1,2]. However, predation risk is often unequal among individuals with different attributes and states within a group. For example, predators may select prey based on their individual vulnerabilities [3–5] to conserve hunting energy or reduce the risk of injury by selecting prey in poor conditions [6–8]. In many taxa, predators disproportionately select young, old, sick, weak, injured, or inexperienced individuals from prey populations [4,9,10].

Spatial positions within the prey group have also been associated with vulnerability to predation [11–13]. Many studies on wild primates have employed the individual identification method, thus contributing to empirical knowledge that different individuals occupy distinct positions within a group. Briefly, individuals that occupy the center of a group may be either dominant [14,15], highly sociable [16], stay longer in the group [17], or female and immature [18,19]. Males can be located in front of the group while travelling, where the risk of encountering predators is high [15,20–22]. However, opposite patterns have been reported in other animals, including primates, where the predation risk is highest in the center [23,24] or rear of the group [21,25,26].

One possible reason for the empirical ambiguity may be related to data collection methods. In previous studies, the spatial positions of individuals were obtained by recording the order in which animals passed through anchor points, such as roads, or by measuring individual distances from the anchor using foot observers. However, individual primates have recently been reported to exhibit varying degrees of intolerance to predator as well as human observer approaches [27]. Specifically, shy individuals tend to avoid human observers, creating a bias in the spatial positioning data. Hunted individuals may have been observed under these conditions. Global Positioning Systems (GPS) can effectively avoid this problem by recording individual positions even in the absence of human observers.

Another reason for the empirical ambiguity with respect to predation risk could be predator hunting styles, which are generally divided into coursing and ambushing. Because coursing predators (primarily canids and hyenas) chase their prey over long distances and attack from open areas, individuals at the edge of a group are more likely to encounter predators than individuals at the center and are thus more vulnerable (‘marginal predation’, Table 1) [12,28]. In contrast, ambush predators (mainly felines) approach their prey unnoticed and randomly appear anywhere in the group; therefore, the predation risk is higher for isolated individuals [29–32]. This indicates that individuals vulnerable to one type of predator may not be as vulnerable to another type of predator. Most studies on predator-prey responses have focused on only one of multiple predator species in an ecosystem [33,34]. Studies that account for the risk of multiple predators should derive a general rule regarding positions vulnerable to predation.

**Table 1:** Summary of advantages and weaknesses of each predation risk proxy, and suggested cases of when to use each depending on the predator’s preferences.

Proxy	Predator’s preference	Advantages	Weaknesses	References
Unlimited domain of danger (UDOD)	Predators attack the edge of the group (one or several individuals)	• Simple “edge” vs. “central” individual definition	• Infinite areas are ecologically unrealistic • Possibly unrealistic measure of predation perception • Distances to and locations of neighbors are unknown	29
Minimum convex polygon (MCP)	Predators attack the strict boundary of the group	• Strict “boundary” vs. “inside” definition	• Distances and locations of neighbors are unknown	39, 40
Limited domain of danger (LDOD)	Predators attack isolated individuals (less neighbors and more widely spaced to them)	• Includes information on the predator’s range of attack	• Difficulty in defining the radius • Possibly unrealistic measure of predation perception • Limited information on the number of neighbors and their closeness (distance)	42
Surrundedness	Predators attack isolated individuals (less surrounded by neighbors)	• Includes information on the position and direction of all other group members • More fine-scale measure of spatial centrality (vs. UDOD and MCP)	• Distance to neighbors unknown	47
Dyadic distances	Predators attack isolated individual (more widely spaced to neighbors)	• Includes information on the distances to all other group members	• Location (direction) of neighbors are unknown	32

In this study, we used multiple GPS devices simultaneously on a group of individually identified wild baboons to re-examine the age, sex, and dominance of individuals in positions considered vulnerable to each of the two hunting styles of terrestrial predators, under conditions that minimized the influence of human observers. Depending on food dispersal, predation pressure, and group size within the environment, the spread of animal groups varies, even within conspecific species [35]. In general, as long-lived and highly social mammals, primates form lasting social bonds and maintain cohesive social groups. Baboons in the wild reportedly prefer four to six specific neighbors [36]. Although the sample size of predation events was small, a previous study reported that predators were more likely to hunt adults than juveniles and males than females [37]. Instead of directly observing predation events, we calculated the individual predation risk using five alternative indices (proxies) that theoretical studies have proposed to predict the predation risk faced by individual prey in two-dimensional space. We tested the following three hypotheses. Hypothesis 1 was that adult males are located peripherally, where coursing predation is most likely to occur. Hypothesis 2 was that isolated individuals vulnerable to ambush predators are adult males. Hypothesis 3 was that dominant individuals, especially the alpha male, occupy the center of the group.

## Methods

### Study site and subjects

This study was conducted at the Mpala Research Centre (0°20’N, 36°50’E) on Laikipia Plateau, Kenya from July 19 to August 18, 2019. The surrounding environment included bushes and riverine woodlands dominated by *Acacia* spp. The annual rainfall at Mpala is approximately 700 mm, with trilateral peaks in April–May, October–November, and July–August [38]. The rainfall was highly variable in each year, and only approximately half (37 mm) of the normal rainfall (56 mm, 1998–2018) occurred in July 2019, which resulted in good visibility for observers.

The wild anubis baboon (*Papio anubis*) ‘AI’ group has been studied since 2011. During the study period, the group of 63 individuals, including infants, comprised 8 adult males and 16 adult females. During the study period, excluding the first five days, we recorded behaviors via *ad-libitum* observation, and the alpha male was determined through direct behavioral observations, such as attacks or displacements of other individuals in the group. Genetic analysis by long term data showed that the alpha male in the study period, *MG*, was born in this group. Leopards, lions, and hyenas were sympatric with the AI group. The predation rate on the AI group from 2011 to 2016 was estimated at 0.06 individual/year [39].

### Collaring methods and GPS-recorded data

Baboons were captured using walk-in traps (1.8 m^3^) baited with maize and placed near the sleeping sites of the groups. Baboons were not fed except during capture. Two criteria were used for capture. First, a GPS weight limit of no more than 3% body weight was adopted. As a result, 29 infants (46.03% of the AI group) were excluded from the study. Second, we excluded pregnant and lactating females and older individuals to avoid adverse health effects of capture and anesthesia. Individuals were chemically immobilized using ketamine (15 mg/kg) and fitted with GPS loggers (e-Obs Digital Telemetry, Gruenwald, Germany). The collars were fitted to 5 adult males, 10 adult females, 6 adolescent males, and 5 juveniles (2 males and 3 females). Collared adults accounted for 62.5% of the total adult population. The age class ‘juvenile’ in this study included both males and females, because there is little sex difference in social development in individuals under four years of age [15]. The collars were equipped with a breakaway mechanism (Advanced Telemetry Solutions, Isanti, MN, USA), which automatically released them at the end of the study.

We measured the positions of individual baboons using GPS during the day (7:00–18:00) for 30 days from July 19, 2019. Beginning on July 24, the number of tracked individuals decreased as several collars were dropped during the study. The priority of this study was to analyze the spatial positions of the largest number of individuals [40]; thus, we analyzed data from July 19 to 23. For 82.1% (1,119 timestamps) of all 3,305 timestamps (calculated as 60 min x 11 h x 5 days), the positions of ≥ 25 individuals were measured simultaneously.

The presence of estrous females may affect the spatial position of males because males stay in close proximity to those females. We recorded female estrous status every morning and evening at the sleeping sites. The number of estrous females during the analysis period was 1.80 ± 1.70 (mean ± SD), which was not significantly different from the value (1.62 ± 0.95) recorded in the remainder of the study period (*t*-test, t = -0.30, n1 = 5, n2 = 34, *p* = 0.78).

### Group spread

At each timestamp, the linear distance between two individuals with GPS in a group was calculated for all combinations; the maximum linear distance between two individuals at a single timestamp was considered the group spread at that time.

### Predation risk proxies

**Fig 1:**
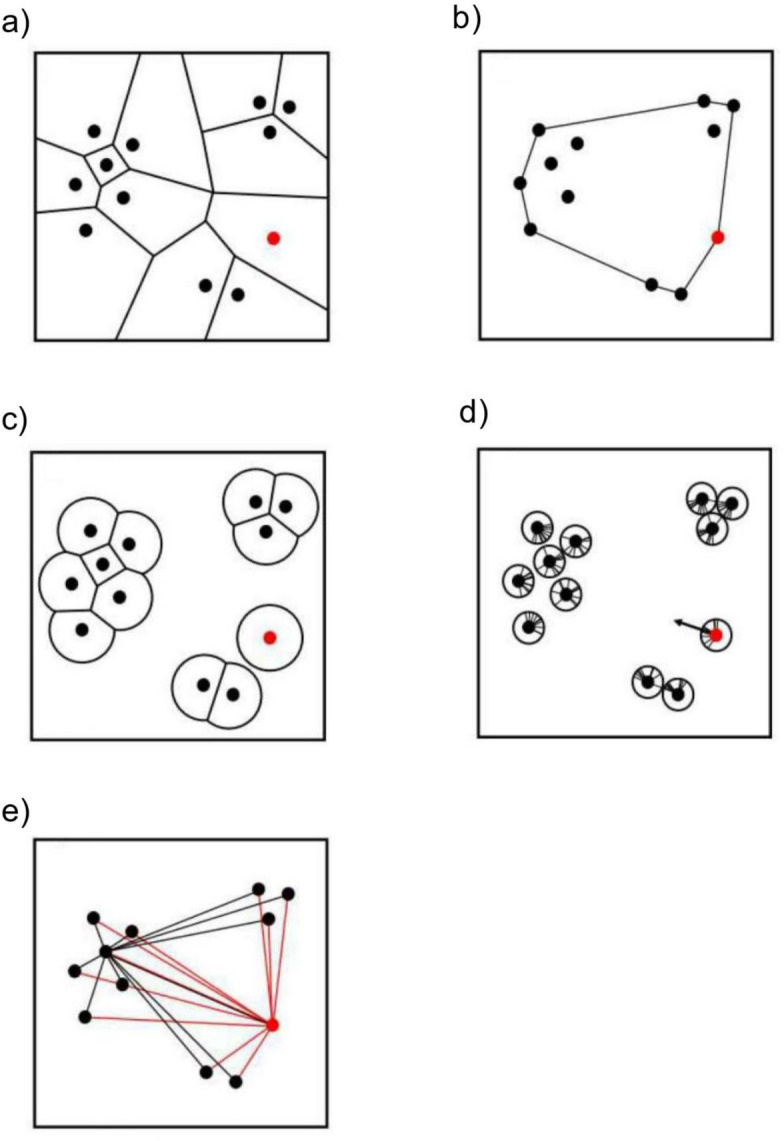
Schematic of each predatory risk proxy. The points represent individuals. a) unlimited domain of danger (UDOD); b) positions based on a minimum convex polygon (MCP); c) limited domain of danger (LDOD); d) surroundedness (circular variance); e) dyadic distances. Points are individuals. If the red point is infinite in the UDOD or on the boundary in the MCP, it is the most vulnerable individual compared with its group mates. Similarly, if the size of the LDOD is maximal, the surroundedness is high, or the dyadic distance is large (the black line is the dyadic distance), the individual in red is the most vulnerable compared to its group mates.

Theoretical studies have proposed several proxies to predict the predation risk at different spatial positions (Table 1 and Fig 1). We used the unlimited domain of danger (UDOD) and minimum convex polygon (MCP) to assess the predation risk for individuals at the edge of the group, where they are most likely to encounter predators (Fig 1A and 1B). The extent to which an individual was surrounded by others was then analyzed using the limited domain of danger (LDOD), surroundedness, and dyadic distances (Fig 1C–1E). The details of each proxy are presented below.

#### a. UDOD

Hamilton [29] proposed the domain of danger (DOD) concept as a measure of the predation risk taken by each prey individual within a group. This concept assumes that a predator currently undetected by prey might appear anywhere within the field, inside or outside the group. DOD is defined as the space around the individual within which a predator can attack and kill it; the larger its size, the higher the individual expected predation risk. The predator is assumed to attack individuals at the closest distance (Table 1). When a perpendicular bisector is drawn on a line connecting points (mother points) placed at arbitrary neighboring positions, the nearest neighbor region formed around each mother point is known as a Voronoi cell. A general problem arises when applying Hamilton’s idea to real animal groups: DOD is defined as a Voronoi cell, but simultaneously, individuals at the edge of the group have biologically unrealistic unbounded Voronoi polygons (UDOD) (Fig 1A).

#### b. MCP

Predation risk is higher for individuals at the edge of a group than for those at the center [41]. One strategy to determine the individuals occupying these positions is based on a MCP. In this method, animals were defined as being at the edge (boundary) of the group if they belonged to the set of vertices describing the smallest convex polygon enclosing all members of the group (Fig 1B) [42]. Consequently, all other individuals were defined as being inside the group.

#### c. LDOD

Applying Hamilton’s idea to real animal groups raises the problem that the DODs of edge individuals are infinite, which is a biologically unrealistic assumption [43]. Therefore, subsequent theoretical studies have propounded an alternative proxy known as LDOD [44]. The LDOD is the area of a circle of radius *r* centered on each animal. This radius corresponds to the range over which a predator can strike, the maximum detection distance of the predator, or the distance within which the predator can successfully launch an attack after approaching undetected [44]. The LDOD of an isolated focal individual is maximal, defined as π*r*^2^, where *r* is the predator’s attack distance (Fig 1C). If there is a conspecific individual within the maximum LDOD, the distance between the two is less than 2*r*, and the created bisector reduces the LDOD.

The definition of *r* based on field reports is important to represent the most realistic range of danger. Information on the attack ranges of the main ambush predators of baboons, leopards, and lions is scarce in the literature, as only two studies can be cited. Observations by Bailey [45] suggest that prey will allow visible leopards to approach up to 25 m without feeling threatened, but it will flee if they are closer than 10 m. Specifically, the distance limit of 10–25 m allows the leopard to hide and stay undetected by a baboon; therefore, 25 m was chosen for this study. Because anubis baboons also suffer from lion predation, we also calculated LDODs with a radius of 70 m based on the study by Cowlishaw [46] on chacma baboons (*Papio ursinus*) and their use of refugia against lions and leopards. The 70-m value is the median distance to a refuge, where chacma baboons living in open plains spend most of their time. This is the hypothesized distance beyond which a baboon is outrun and captured by a predator before reaching safety. Empirically, Stander [47] reported that 73% of lion kills had short chase distances of less than 20 m, whereas the remaining 27% were between 20–150 m. Moreover, Scheel [48] reported hunting distances of up to 200 m. In this study, individual LDODs can theoretically vary between a minimum of 0 m^2^ (if at least two individuals share the same location) and a maximum of 1,960 and 15,369 m^2^ (the individual is isolated), with radii of 25 and 70 m, respectively.

#### d. Surroundedness

‘Surroundedness’ refers to the degree to which an individual is surrounded by all group members, with the expectation that more surrounded individuals are less vulnerable to predation. This study assessed surroundedness using circular variance, a measure based on circular statistics [49].

The following calculations were performed according to Cremers and Klugkist [50]. A unit was drawn around a focal individual, and the directions of all group members were projected as points on the circle’s circumference. Connecting these points to the origin (the focal individual) yielded individual vectors (Fig 1D). Vectors with the same value were stacked in the radial direction of the base circle. The resulting vector (R) was created by connecting the toe of the first vector to the head of the last vector. Relative to the focal individual, the direction indicated where most other group members belonged.

In this study, we calculated circumpolar variance, defined as 1-$\bar{R}$. The length of the mean resultant vector ($\bar{R}$) is R divided by the number of created vectors. A variance of 1 means that the individual is surrounded by many individuals, whereas a variance of 0 means that the individual is not surrounded; that is, all other members are located in one particular direction.

#### e. Dyadic distances

Predation risk will likely vary with the number and proximity of conspecifics through effects such as dilution and accelerated predator detection [32]. Thus, one way to determine which individuals are more susceptible to predation is to determine their distance from all members. Individuals that maintain a greater distance from others are more physically isolated (Fig 1E) and thus may be the preferred target for predators. Therefore, we calculated the Euclidean distance for each focal individual and the dyadic combination of all other group members at each timestamp.

### Statistical analyses

We aimed to explore the potential differences among age-sex classes for all predation risk proxies. Because LDODs and surroundedness are continuous variables, we used linear mixed models (LMMs). The autocorrelation structure of the measured sample points was analyzed, and the data were fitted using a Gaussian model with the following equation:

$$Semi-variance\;(\tau )=\;c\;[1-\exp\;(-\tau^{2}/a^{2})],$$

where c, a, and τ represent sill, range, and time-lag (min), respectively. These calculations were conducted at each timestamp (every 1 min interval) using the Python 3 (version 3.7.10) libraries NumPy (version 1.20.2), Pandas (version 1.3.5), and Matplotlib (version 3.3.4). We set these variables as the response variables, with age-sex classes (factors with four levels: adult males, adult females, adolescent males, and juveniles) as predictors. The interaction term between age and sex was also included. Because both the UDODs and MCP positions are binomial factors, we used generalized linear mixed models (GLMMs). Both models were fitted using a binomial error structure, with the individual spatial position as the response variable. The UDOD was defined as finite UDOD [0] vs. infinite UDOD [1], and the MCP positions were inside [0] vs. boundary [1]. The predictors were similar to those of the LMMs. To study the differences in dyadic distances between each age-sex class, we used an LMM with dyadic distances as the response variable and pairs of age-sex classes (factors with ten levels) as the predictor. A two-sided test was used to ascertain the differences in mean values for each age-sex group. All LMMs and GLMMs were followed by Tukey’s post-hoc comparisons with Bonferroni correction to assess the specific differences between each age-sex class.

In all models, individual identities were set as random factors on the intercept. The significance of predictors in each model was computed with the model including the predictor against the model excluding the predictor (Wald χ^2^ for LMMs and likelihood-ratio χ^2^ tests for GLMMs, Anova Type III). Marginal R^2^ and conditional R^2^ were given for each model, representing the variance explained only by fixed effects and the variance explained by fixed and random effects, respectively. Results were considered significant at the α = 0.05 level for the models and post-hoc comparisons.

R packages {version 4.1.1; R Core Team [51]} used for the analyses were ‘lme4’ for running the LMMs and GLMMs, ‘multcomp’ for the post-hoc analyses, ‘car’ for ANOVA tables, and ‘MuMin’ for R2 computation.

## Results

### Group spread

The median group spread across all timestamps was 205.18 m (136.20 m in the first quartile and 345.18 m in the third quartile). The maximum and minimum distances were 3,560.79 and 20.89 m, respectively.

### Risk of exposure to predation

#### a. UDODs

**Fig 2:**
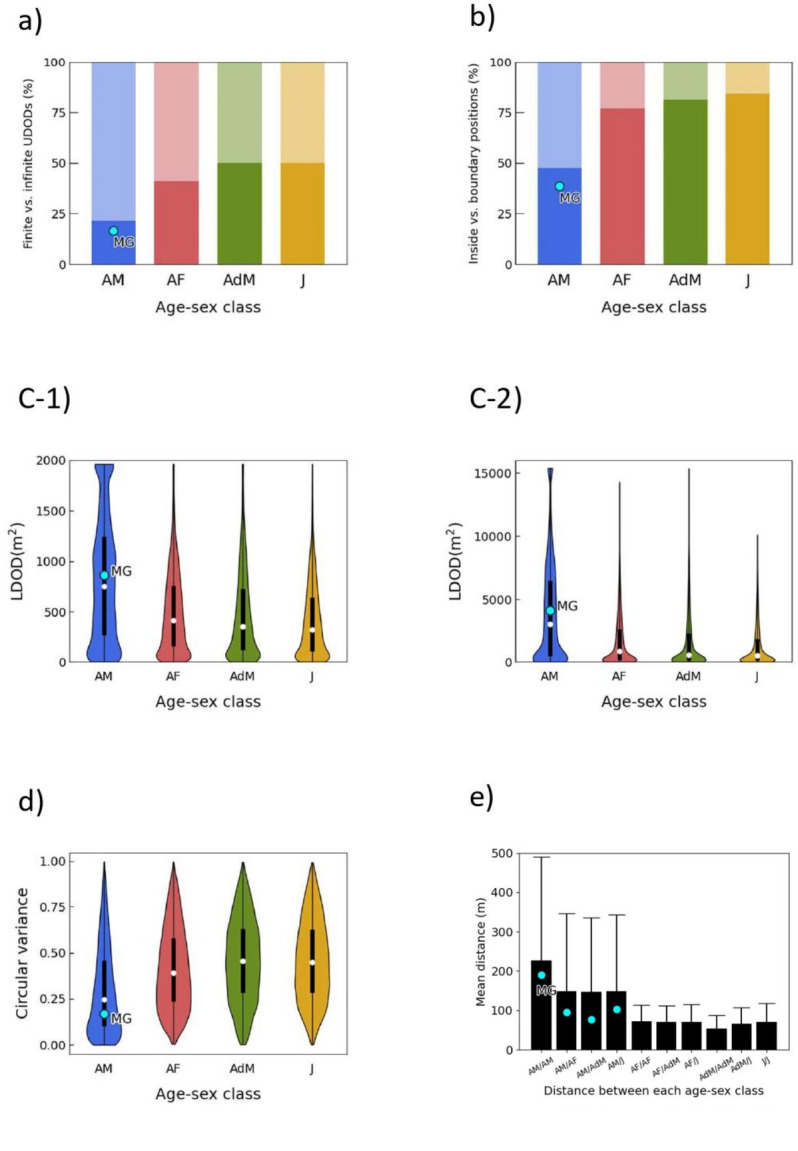
Calculated results for each predation risk proxy. a) Percentages of finite (dark gray) vs. infinite (light gray) UDODs for each age-sex class, ordered from lowest to highest. Age-sex class abbreviations are as follows: AM = adult males, AF = adult females, AdM = adolescent males, and J = juveniles. Notation is similar in subsequent figures. b) Percentages of the inside (dark gray) vs. boundary (light gray) MCP positions for each age-sex class. c-1, c-2) Violin plots along boxplots of LDODs. c-1) radius of 25 m and c-2) radius of 70 m. ⚬ denotes the mean value and ⚫ denotes the alpha male value. d) Violin plots along boxplots of surroundedness. e) Mean distance (m) between each age-sex class. Bars are mean associated with their standard error.

Infinite UDODs were most common among adult males, followed by adult females, adolescent males, and juveniles (Fig 2A and S1 Table). Significant differences were observed in the percentage of finite UDODs among the age-sex classes (χ^2^ = 50.40, *p* < 0.001). Adult males had the smallest proportion of finite UDODs (vs. adult females, z = 4.63, *p* < 0.001, vs. adolescent males, z = 5.87, *p* < 0.001, vs. juveniles, z = 6.33, *p* < 0.001). No significant differences were observed in any other comparisons.

#### b. MCP

Significant differences were observed in the percentage of individuals at the edge/center among the age-sex classes (χ^2^ = 83.52, *p* < 0.001, Fig 2B and S2 Table). Adult males were more frequently on the edge than individuals of other age-sex classes (vs. adult females, z = 7.36, *p* < 0.001, vs. adolescent males, z = 6.86, *p* < 0.001, vs. juveniles, z = 8.04, *p* < 0.001). No significant differences were observed in any other comparisons.

#### c. LDODs

There were differences among the age-sex classes for both LDODs with radii of 25 m (attack of a leopard) and 70 m (attack of a lion; Fig 2C). For LDODs with a radius of 25 m, the percentages of finite LDODs among the four age-sex groups significantly differed (χ^2^ = 606.76, *p* < 0.001, S3 Table). Adult males had the largest LDODs (vs. adult females, z = 18.67, *p* < 0.001, vs. adolescent males, z = 19.75, *p* < 0.001, vs. juveniles, z = 22.36, *p* < 0.001). Similar qualitative differences were found for the 70-m radius LDODs (χ^2^ = 41.93, *p* < 0.001, vs. adult females, z = 4.94, *p* < 0.001, vs. adolescent males, z = 5.22, *p* < .001, vs. juveniles, z = 3.91, *p* < 0.001). No differences were found at either 25 or 70 m in any of the other comparisons.

#### d. Surroundedness

The degree of surroundedness by group members showed significant differences among the age-sex classes (χ^2^ = 343.16, *p* < 0.001, Fig 2D and S4 Table). Adult males had the smallest circle variances (vs. adult females, *t* = 13.86, *p* < 0.001, vs. adolescent males, *t* = -16.16, *p* < 0.001, vs. juveniles, *t* = -15.80, *p* < 0.001). No significant differences were observed in any other comparisons.

#### e. Dyadic distances

Of all age-sex classes, the mean distance between adult males was the greatest, and that between adolescent males was the smallest (Fig 2E). Distances between adult males (mean ± SE, 226.38 ± 263.38 m) were significantly longer than those between adult females (71.45 ± 42.01 m, z = 3.31, *p* < 0.005, S5 Table) or those between adolescent males (53.66 ± 33.98, z = 3.30, *p* < 0.005). However, no significant differences were found for the distance between adult males and that between juveniles (70.83 ± 46.51, z = 2.73, *p* > 0.005).

### The special position of the alpha male

The UDOD and MCP results showed that the alpha male, *MG*, was located peripherally to the group to a similar extent as the other males (Fig 2A and 2B). In addition, LDOD, surroundedness, and dyadic distance metrics indicated that *MG* was slightly more spatially isolated than the average for adult males.

## Discussion

In Hypothesis 1, we proposed that adult males are located peripherally, where coursing predation is most likely to occur. Our UDOD and MCP results showed that, as expected, adult males were located more peripherally, consistent with previous studies on primates [52] and other animals [53–55]. Two well-studied hypotheses have been proposed from ecological and sociological perspectives to explain why males are located peripherally. One hypothesis involves a trade-off between increased foraging efficiency and higher predation risk [54,56]. Adult males with larger bodies and higher nutritional requirements may have prioritized greater food accessibility over predation risk and occupied peripheral positions. In addition, adult males may play a role in protecting females and their young in the center of the group through vigilance, alarm, mobbing, and counterattacks against predators or neighboring groups [57]. Four reasons have been cited for this behavior: paternal investment, maintenance of male and female social bonds, group enhancement benefits, and quality signaling in the mating market [58].

Hypothesis 2 states that adult males are the most isolated individuals vulnerable to ambush predators. As expected, the LDOD, surroundedness, and dyadic distance indicated that adult male baboons were the most isolated. One reason for this may be the social structure of baboons. In all four savanna baboon species, anubis, yellow, chacma, and guinea baboons, females remain in their natal groups throughout their lives, whereas males generally move to another group after sexual maturity. In species with male dispersal, adult males are expected to have few or no relatives in the postnatal group except for their offspring. DNA microsatellite association analyses in some primates have reported that adult males have few close relatives in their postnatal groups [59,60]. As unrelated males are severe competitors for females in estrus, adult males maintain a long distance from each other to escape attacks by other males and serious injuries [61].

Hypothesis 3 states that individuals with high social status among adult males, especially alpha males, occupy the secure core of the population. Dominant individuals, who can access preferential foods even under intense competition, are expected to be in the safe center of the group [31,62–64]. However, the alpha male in this study was peripheral and isolated, as were other males. Intimacy in the male-female relationship, known as ’friendship’ in baboons [65], prevents male isolation, and this relationship forms through the offspring in yellow baboons [66]. One possible explanation for our result may be that the alpha male, although born in this group, had been in this position for only approximately one year and had few female friends and offspring.

We addressed the methodological and theoretical advantages and disadvantages of the proxies used in this study for each predator hunting style. A strong assumption involves variation in predation risk based only on spatial positioning, for which predators can show variable preferences. First, assuming a coursing predator, UDOD and MCP positions are similar in that they use a simple dichotomy to define individuals located on the edge of the group, as opposed to centrally positioned ones. However, both methods vary in their definition of edges owing to their underlying mathematics, which can result in slightly different results. For example, Voronoi diagrams can define more individuals as peripheral (‘edge’ individuals) compared to MCP vertices that only consider peripheral individuals as those strictly forming the spatial boundary of the group; individuals with finite UDODs are always inside the group. These differences in definition are illustrated by the left and top-right clusters of individuals in Fig 1A and 1B, respectively. Use of one proxy will then depend on whether the predator prefers to strictly attack the individual forming the boundary of the group or any individual forming the periphery (‘edge’), including the one at the strict boundary. Both methods are useful only when predators consistently attack the edge of a group (marginal predation). Indeed, this has been observed in different species [29,67–69].

From a theoretical perspective, LDODs are based on the notion that animals react to the open spaces around them, whereas the surroundedness and dyadic distances highlight the possible effects of several neighbors. In a context where predators do not consistently attack peripheral individuals, but choose isolated individuals, i.e., those outside or inside the group who are relatively distant from group members, other variables may be more pertinent. LDODs can be useful, because they include information on the distance to neighbors regardless of the individual position within the group. However, their calculation can be complicated if the group has several types of predators with different hunting techniques. Consequently, this raises the question whether one should expect changes in results when comparing groups of different radii. As such, the radius rests on a detailed description of predator-prey interactions and is somewhat arbitrary.

Considering that LDODs only include information on the closest neighbors, the surroundedness and dyadic distances complement each other and provide a better measure of isolation based on the positions of group members. Surroundedness is a more fine-scale measure of spatial centrality/peripherality within a group (especially compared to UDODs and MCP positions) and includes information on the position and direction of all other group members. Dyadic distances are a more fine-scale measure of neighbor spacing and include information on the distance to all other group members. In some species, such as straggler fish, isolation, rather than the periphery, is correlated with higher predation risk; i.e., isolated individuals outside the school, where centrally positioned members are also more at risk [23]. Similar patterns could be observed for redshanks, where more widely spaced individuals are preferred targets for predators compared to their nearest neighbors [70].

The number of collared individuals is the most limiting factor in emphasizing these predation risk proxies and any other proxies correlated with predation risk. Missing (non-collared) individuals influence the shape of the Voronoi diagrams and MCPs, change the size of the LDODs, and modify the circular variance and distances between individuals. Because predation risk is always related to all other group members, the location of the maximum number of, if not all, individuals must be as accurate as possible. However, this study prioritized the maximum number of individuals, which has the disadvantage of reducing the number of data days. Farine et al. [30], who conducted similar data collection and analysis, noted that individuals showed a consistent pattern of spatial positioning within the population over many days. However, in the present study, the maximum h-spread of the group was slightly greater than 3 km, which was attributed to an older male lagging behind the group by approximately half a day. Excluding the data from this day, the median and maximum spread distances for the group were 184.83 and 734.11 m, respectively. Because reducing the number of days of analysis by a factor of h can cause such variation, a methodology must be developed to determine the time interval of positioning suitable for spatial location analysis and the number of valid data days.

Elucidating the costs of predation risk associated with individual spatial positions within a group is important for understanding group formation and maintenance. The current study showed that adult males consistently occupied vulnerable positions to predators that would attack our ambush them from outside or within the group, respectively. Determining whether adult males position themselves on the edge voluntarily or unwillingly is difficult; however, it is an interesting future challenge for both ecological and social research.

## Supporting information

S1 Table Summary statistics of USOD. (PDF)

S2 Table Summary statistics of percentages of boundary positions. (PDF)

S3 Table Summary statistics of LODO size with radii of 25 m and 70 m. (PDF)

S4 Table Summary statistics of surroundedness. (PDF)

S5 Table Summary statistics of mean distance. (PDF)

S1 Data (ZIP)
