# Supplementary material for: Estimating individual exposure to predation risk in group-living baboons, Papio anubis
Source: PLoS One. 2023 Nov 8;18(11):e0287357. doi: 10.1371/journal.pone.0287357 (PMC10631679; doi:10.1371/journal.pone.0287357)
Supplement: S1 Table — (PDF) [file pone.0287357.s001.pdf]

Supplementary Material

S1

Top: Percentages of infinite positions are given for UDODs.

Bottom: Results of a generalized liner mixed model (GLMM). The χ^2 and the p values associated from the likelihood-ratio χ^2 test of the comparison between the model with age-sex predictors and the model without them is given (analysis of variance type III). For the age-sex factor, adult males are the reference level. Marginal $R^2 = 0.04$, Conditional $R^2 = 0.07$, variance of the random effect: $\sigma = 0.12$, SD = 0.34.

	AM	AF	AdM	J
Infinite UDOD	78.70	62.43	53.49	52.11

	β	SE	df	χ^2	p value
Intercept	1.34	0.14			
Age-sex			3	50.40	< 0.001
AF	-0.82	0.18			
AdM	-1.17	0.20			
J	-1.27	0.20			
