# Supplementary material for: Estimating individual exposure to predation risk in group-living baboons, Papio anubis
Source: PLoS One. 2023 Nov 8;18(11):e0287357. doi: 10.1371/journal.pone.0287357 (PMC10631679; doi:10.1371/journal.pone.0287357)
Supplement: S2 Table — (PDF) [file pone.0287357.s002.pdf]

S2

Top: Percentages of boundary positions.

Bottom: Results of the GLMM. Marginal $R^2 = 0.07$, Conditional $R^2 = 0.09$, variance of the random effect: $\sigma = 0.15$, SD = 0.38.

	AM	AF	AdM	J
Boundary	52.47	24.43	20.01	16.26

	β	SE	df	χ^2	p value
Intercept	0.10	0.15			
Age-sex			3	83.52	< 0.001
AF	-1.29	0.18			
AdM	-1.48	0.21			
J	-1.77	0.22			
