# Supplementary material for: Estimating individual exposure to predation risk in group-living baboons, Papio anubis
Source: PLoS One. 2023 Nov 8;18(11):e0287357. doi: 10.1371/journal.pone.0287357 (PMC10631679; doi:10.1371/journal.pone.0287357)
Supplement: S3 Table — (PDF) [file pone.0287357.s003.pdf]

S3

Top: Summary statistics of LDOD size with radii of 25 m and 70 m. Numbers given are mean ± SD (min – max).

Bottom: Results of linear mixed models (LMMs) with autocorrelation. LDODs with 25 m: Marginal R^2 = 0.09, Conditional R^2 = 0.09, variance of the random effect after Box-Cox conversion: σ = 8.80, Intercept = 3.64E-07. LDODs with 70 m: Marginal R^2 = 0.14, Conditional R^2 = 0.14, variance of the random effect after Box-Cox conversion: σ = 4.74, Intercept = 1.00.

	AM	AF	AdM	J
r = 25 m	818.03 ± 608.18<br>(0.53 – 1960.34)	500.35 ± 410.88<br>(0.36 – 1960.34)	475.14 ± 431.78<br>(0.31 – 1960.34)	416.79 ± 369.04<br>(0.51 – 1960.34)
r = 70 m	4027.91 ± 3487.28<br>(0.44 - 15393.02)	1075.34 ± 2142.46<br>(0.31 – 15359.81)	1573.15 ± 2142.46<br>(0.31 – 15359.81)	1251.31 ± 1642.62<br>(0.51 – 10115.96)

r = 25 m	β	SE	df	χ^2	p value
Intercept	817.59	12.00			
Age-sex			3	707.78	< 0.001
AF	-317.53	14.67			
AdM	-342.68	16.30			
J	-401.225	16.94			

r = 70 m	β	SE	df	χ^2	p value
Intercept	4026.77	69.32			
Age-sex			3	1040.90	< 0.001
AF	-2322.38	85.76			
AdM	-2451.77	95.30			
J	-2777.47	99.04			
