# Supplementary material for: Estimating individual exposure to predation risk in group-living baboons, Papio anubis
Source: PLoS One. 2023 Nov 8;18(11):e0287357. doi: 10.1371/journal.pone.0287357 (PMC10631679; doi:10.1371/journal.pone.0287357)
Supplement: S4 Table — (PDF) [file pone.0287357.s004.pdf]

S4

Top: Summary statistics of Surroundedness. Numbers given are mean ± SD (min – max).

Bottom: Results of a linear mixed model with autocorelation. Marginal R^2 = 0.06, Conditional R^2 = 0.06, variance of the random effect after Box-Cox conversion: σ = 0.33, Intercept = 2.95E-05.

	AM	AF	AdM	J
Surroundedness	0.30 ± 0.24<br>(0 – 1.00)	0.42 ± 0.22<br>(0.004 – 1.00)	0.46 ± 0.22<br>(0.002 – 1.00)	0.46 ± 0.22<br>(0.002 – 1.00)

	β	SE	df	χ^2	p value
Intercept	0.30	0.008			
Age-sex			3	299.34	< 0.001
AF	0.12	0.01			
AdM	0.16	0.01			
J	0.16	0.01			
