# Supplementary material for: Estimating individual exposure to predation risk in group-living baboons, Papio anubis
Source: PLoS One. 2023 Nov 8;18(11):e0287357. doi: 10.1371/journal.pone.0287357 (PMC10631679; doi:10.1371/journal.pone.0287357)
Supplement: S5 Table — (PDF) [file pone.0287357.s005.pdf]

S5

Top: Mean distances (m) and their standard-errors of each pair of age-sex classes.

Bottom: Table reporting all comparisons of mean distances (m) and their standard-errors between each pair of age-sex classes. Signs of mean distances reported correspond to the mean distance between age-sex classes in the left column minus the mean distance between age-sex classes in the top line. Significant mean distances are reported in bold with ** p < 0.01 and * p < 0.05. Non-significant values are left as is. The significance code reported here refer to post-hoc comparisons (with Bonferroni's correction) after conducting the linear mixed model (see Material and Methods section).

	AM/AM	AM/AF	AM/AdM	AM/J	AF/AF	AF/AdM	AF/J	AdM/AdM	AdM/J	J/J
spread mean ±SE (m)	226.38 ± 263.38	148.56 ± 197.79	146.41 ± 189.40	147.93 ± 195.74	71.45 ± 42.01	70.15 ± 40.96	70.51 ±43.87	53.66 ± 33.98	66.10 ± 41.65	70.83 ± 46.51

	AM/AM	AM/AF	AM/AdM	AM/J	AF/AF	AF/AdM	AF/J	AdM/AdM	AdM/J	J/J
AM/AM		92.94 ±37.80*	94.21 ± 41.30	93.99 ± 42.9	172.00 ± 51.90	171.51 ± 54.5	171.40 ± 55.70	188.38 ± 57.00*	175.81 ± 58.20	167.09 ± 61.20
AM/AF			2.28 ± 33.00	1.05 ± 35.00	79.06 ± 35.60	78.57 ± 48.50	78.46 ± 49.90	95.44 ± 51.40	82.87 ± 52.70	74.15 ± 55.90
AM/AdM				-1.22 ± 38.70	76.79 ± 48.50	76.29 ± 35.60	76.18 ± 52.60	93.16 ± 39.40	80.60 ± 55.20	71.87 ± 58.3
AM/J					78.01 ± 49.90	77.52 ± 52.60	77.41 ± 35.60	94.39 ± 55.20	81.82 ± 39.40	73.10 ± 43.60
AF/AF						-0.49 ± 33.00	-0.61 ± 35.00	16.38 ± 47.10	3.81 ± 48.50	-4.92 ± 52.00
AF/AdM							-0.11 ± 38.70	16.87 ± 33.60	4.30 ± 51.20	-4.42 ± 54.60
AF/J								16.98 ± 51.20	4.41 ± 33.60	-4.31 ± 38.50
AdM/AdM									-12.57 ± 38.70	-21.29 ± 57.10
AdM/J										-8.72 ± 42.00
J/J										
